# Global Health Teaching in India: A Curricular Landscape

**DOI:** 10.3389/fpubh.2017.00259

**Published:** 2017-09-27

**Authors:** Sanghamitra Pati, Rajeshwari Sinha, Meely Panda, Sandipana Pati, Anjali Sharma, Sanjay Zodpey

**Affiliations:** ^1^Department of Health Research, Regional Medical Research Centre (ICMR), Bhubaneswar, India; ^2^Independent Researcher, New Delhi, India; ^3^Jamia Hamdard University, New Delhi, India; ^4^Department of Health and Family Welfare, Government of Odisha, Bhubaneswar, India; ^5^Public Health Foundation of India, Gurugram, India

**Keywords:** global health, international health, public health education, curriculum, India

## Abstract

Today, health has transcended national boundaries and become more multifaceted. Global health has evolved as a new paradigm and is recently being identified as a thrust area now in India. Despite an existing need for a standardized global health curriculum, there is little information available on its education and curriculum in medical and health education space. In the Indian context, we are yet to have a fuller picture of the current status, including, content, structure, selection, teaching methods of global health, and how students are evaluated in India. The objective of this study was to map courses relating to studies on global health in India and analyze its mode of delivery. A detailed Internet search was carried out to identify global health courses and analyzed for: (i) whether global health is a part of the teaching curriculum, (ii) mode of teaching, (iii) broad contents, (iv) instructional formats, (v) assessment, and (vi) selection process. It was found that delivery of global health education in India was fragmented with limited focus at the undergraduate and postgraduate levels. Global health teaching was largely based on certificate courses or online courses, with hardly any institutions imparting a distinct global health education program. There is also no definite specification as to which institutes can impart teaching on global health education and what the specific eligibility requirements are. Our analysis suggests that efforts should be directed toward integrating global health education into broader public health curriculum. At the same time, the need for generation of global health leaders, creation of a common forum for addressing merits and demerits of global health issues, as well as creation of more opportunities for placements are recognized.

## Introduction

In recent years, the world has made rapid progress toward economic globalization, populations have become more multicultural and borders are being traversed easily. Health issues have therefore moved beyond communities and countries. Increased migration, international travel, commodity and food supply globalization, climate change, reemerging disease patterns and rising burden of chronic diseases, have caused health to transcend national boundaries and become more multifaceted. The concept of health is therefore no longer restricted to disease based and clinical interventions, but has evolved further to assume global dimensions (1).

With the above evolution, the scope of population health has broadened to accommodate two of the new emerging domains of public health–international and global health. Public health is usually viewed as having a focus on the health of the population within a specific country or community (2). International and global health, though often used interchangeably, are different entities. International health differs from public health in focusing on health issues outside of one’s own country. In Koplan et al.’s view, international health caters to health issues, especially infectious diseases, and maternal and child health in low-income countries (2). Global health, on the other hand is not just limited to the location of problems, but focuses on the wider health determinants, and transnational health issues. Koplan et al. also proposed a working definition of global health as “an area or study, research, practice that places a priority on improving health and achieving equity in health among everyone worldwide” (2).

As increased awareness and growing interest on the new paradigm of global health takes place, the need for a standardized global health curriculum in medical education is also evident. Presently, there is limited knowledge on what should actually be the component of an appropriate global health training course to fill an existing gap in medical education. Initiatives to introduce global health discipline as an essential component of the medical education curriculum in developed countries have been made. However, despite the burden of preventable disease being largely predominant in low- and middle-income countries, little information is available on global health related education in these countries.

In the Indian context, global health is yet at its nascent stage and has only been recently identified as a thrust area. We are yet to have a fuller picture of the current status, including, course content, structure, selection, teaching methods of global health, and how students are evaluated in India. At the present policy level also, there is no focus on promoting global health as a part of regular medical curriculum (3). The objective of the present work is to map courses related to teaching of global health in India and analyze its mode of delivery and implications on policy. It is expected that the results would provide guidance and critical inputs for strengthening teaching on global health at multiple levels.

## Methodology

To obtain the best available insights into the imparting of global health teaching in India, an iterative process was adopted. A detailed Internet search was carried out to identify global health courses using the Google search engine with “global health, global public health, international health, global issues, and universal health” as the key words. The websites of Association of Indian Universities, Indian Council of Medical Research, University Grants Commission, Medical Council of India, Dental Council of India, Indian Nursing Council, and Ministry of Health and Family Welfare were searched (3). A similar search in the websites of the Indira Gandhi National Open University (IGNOU), professional associations, namely, Indian Medical Association, Academy of Family Physicians of India, World Association of Family Doctors (WONCA), World Health Organization (WHO), and various public health institutes, corporate hospitals, and autonomous medical institutes, such as All India Institute of Medical Sciences (AIIMS), Christian Medical College (CMC), Vellore, Post Graduate Institute (PGIMER), Chandigarh, and Jawaharlal Institute of Post Graduate Medical Education and Research (JIPMER), Puducherry, were also carried out in October 2015. The search was limited to courses/programs offered in India and also to collaborations between Indian and foreign institutes, if any. Detailed information about the courses was collected from the institutions’ designated websites as well as through mail and telephonic contacts. Short-term courses spanning few days—few weeks, seminars, and workshops were excluded. To examine the extent of global health teaching within the ambit of health professional education or any other courses, a thorough scan of respective curricula was also performed. The syllabi of community medicine in undergraduate medical, dental, nursing, and allied health sciences were analyzed to map the specific content. Further, masters/diploma in public health and management programs were also examined to identify global health teaching, if any.

We analyzed the course for: (i) whether global health is a part of the teaching curriculum, (ii) the mode of teaching, (iii) the broad contents, (iv) the instructional formats or methods used to teach, (v) assessment, if any, and (vi) the students’ selection process. The specification on where, how, and what is taught was summarized and compiled into a matrix. To compare and contrast the existing global health education framework in India to that of the global landscape, an Internet search was conducted, and list of renowned institutes offering global health education worldwide was collected. The course contents were retrieved, analyzed for the main domains being dealt with, and then compared those being offered by the Indian institutes.

## Results

The global health courses being offered in India are shown in Tables 1 and 2. We see that global health education, as well as teaching, in India is currently at its nascent stage and is gradually growing. There are not many courses currently being offered as main domains. The Centre for International Politics, Organization and Disarmament (CIPOD), is one of the oldest Centres of the School of International Studies, which offers MPhil and PhD on various courses related to international affairs along with global health issues and their management. The global and public health program in Manipal University covers the international aspects of environmental health, maternal and child health, infectious diseases, epidemiology, and health policies. It includes weekly field-based practicum and implementation of various WHO programs at respective levels. The Global Institute of Medical Sciences (GIMS), Baroda and Indian Institute of Tourism and Travel Management (IITTM), Gwalior provide management courses, whereas Indian Institute of Health Management Research (IIHMR), Jaipur and Indian Institute of Public Health and Hygiene (IIPHH), New Delhi provide public health courses where global health forms an interlinking component. Whereas the former two institutes have provisions to take graduates in any discipline for the course, the latter selects only healthcare professionals. Courses are jointly offered by distinguished faculty from Johns Hopkins Bloomberg School of Public Health and IIHMR, who are involved in public health locally, nationally, and internationally. There are many other schools of international studies which mainly cater to global politics, public administration, and legal issues. These have not been included in the matrix since global health as such is not their domain. Online courses with global/international health as the main domain are provided presently by Public Health Foundation of India (in collaboration with SDSN edu) and Apollo Medvarsity IPPC.

**Table 1:** Global health courses being offered in India: location, domain, certification, duration, and fee.

Center	Location	Domain	Certification	Duration	Fee
**Courses with global health as the main domain**					

Manipal University	Karnataka	International affairs and public health	Degree in global and public health program	1 year	Rs. 2.5 lakhs

CIPOD, Jawaharlal Nehru University	New Delhi	International issues, health, globalization, economy, politics, and relations	MPhil, PhD, MA	2–4 years	Within Rs. 5,000

**Courses with global health as a part of the main domain**					

GIMS	Baroda, Gujarat	Global health, acts, management, metrics, and assessments	PG Diploma in Public Health Management	1 year	Rs. 13,000

GIMS	Baroda, Gujarat	Global issue, travel issues, policies, and management	Diploma in Medical Tourism	6 months	Rs. 10,000

IITTM	Gwalior, Madhya Pradesh	Medical tourism, travel, international business, and issues	Post Graduate Diploma in Management	2 years	Rs. 2.5–3 lakhs

IIPHH	New Delhi	Public health and education	Diploma in Public Health	1 year	–

CMC, Vellore	Tamil Nadu	Global health issues and policies	Masters in Family Medicine	2 years	Rs. 90,000

IIHMR, Jaipur in association with John Hopkins University	Jaipur	Real world situations in public health and its management	Certificate for Master’s in Public Health	1.5 years	–

**Online courses with global/international health as the main domain**					

Public Health Foundation of India in collaboration with SDSN edu	New Delhi	Global public health	Digital Certificate on Global Public Health	11 weeks	Open course and free

Apollo Medvarsity IPPC	Apollo Hospital, Hyderabad	Global best practices related to child health	Certificate in International Postgraduate Pediatrics	1 year	–

**Table 2:** Global health courses being offered in India: correspondence, eligibility, seats, and affiliation.

Center	Correspondence	Eligibility	Seats	Affiliated to
**Courses with global health as the main domain**				

Manipal University	admissions@manipal.edu	Graduate	–	Manipal University

CIPOD, Jawaharlal Nehru University	directoradmissions@mail.jnu.ac.in	Any graduate	–	Jawaharlal Nehru University

**Courses with global health as a part of the main domain**				

GIMS	gimsindia2010@gmail.com	Graduates, MBBS, BHMS, social science, BPT, etc.	40	Global Educare Trust, New Delhi

GIMS	gimsindia2010@gmail.com	10 + 2 graduate	40	Global Educare Trust, New Delhi

IITTM	iittm@sancharnet.in	Bachelor’s degree (10 + 2 + 3) in any discipline	93	AICTE, AIU

IIPHH	iphhadmission2013@gmail.com	Health workers and professionals	–	Director of Technical Education and Training, Govt. of NCT Delhi

CMC, Vellore	dedu@cmcvellore.ac.in	Medical Council of India recognized MBBS	100	CMC, Vellore

IIHMR, Jaipur in association with John Hopkins University	iihmr@iihmr.edu.in	Doctoral degree/bachelors degree with high quality experience	–	IIHMR

**Online courses with global/international health as the main domain**				

PHFI in collaboration with SDSN edu	gphmooc@phfi.org	Health care professionals	Online	PHFI
				SDSN edu

Apollo Medvarsity IPPC	apollohealthcity@apollohospitals.com	MBBS	Online	AHERF and Medvarsity

CIPPOD, Centre for International Politics, Organization and Disarmament; GIMS, Global Institute of Medical Sciences; IPPC, International Postgraduate Pediatric Certificate; AHERF, Apollo Hospitals Educational and Research Foundation; CMC, Christian Medical College; IIHMR, Indian Institute of Health Management Research; PHFI, Public Health Foundation of India; IITTM, Indian Institute of Tourism and Travel Management; IIPHH, Indian Institute of Public Health and Hygiene. AICTE, All India Council for Technical Education; AIU, Association of Indian Universities

*–Indicates that exact data could not be retrieved*.

**Figure 1:**
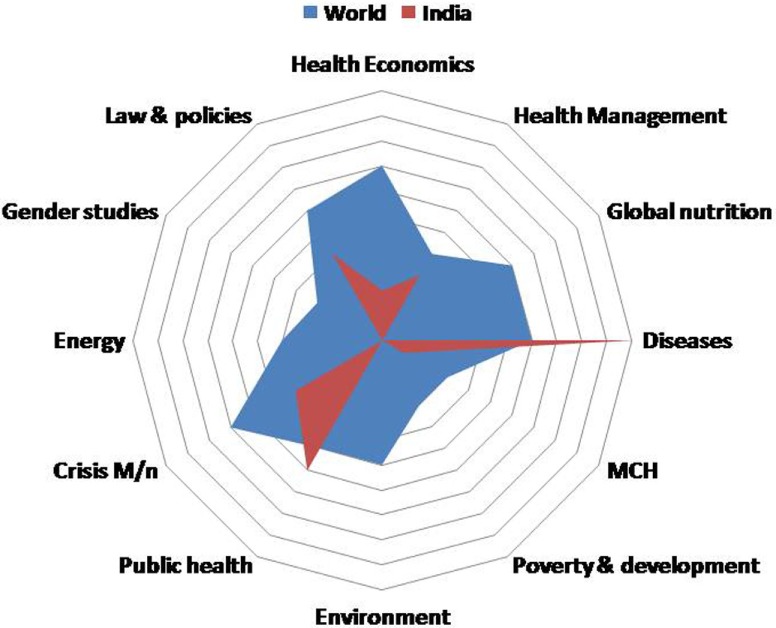
Radar diagram for comparison of topics being covered under global/international health, worldwide, and in India.

Further, we made an effort to collect the topics being covered as part of the curriculum in the 10 best ranked institutes of the world as per QS World University rankings, having separate departments for global affairs and imparting global health education. A radar diagram was used for comparison of the global and the Indian scenario (Figure 1). An overview of the radar diagram shows that these institutes mostly cater to teaching the topics on health systems, health economics, global nutrition, public health; maternal and child health; global infectious and non-communicable diseases, humanitarian studies, population ethics, international security, cultural policies, global governance, migration, human development, poverty, environment, synthetic biology, immunology, emergencies, and disaster management. It was found that, Indian institutes mostly cater to topics such as diseases, public health, crisis management, and legal issues. However, these global institutes do have collaborations with India and have taken up many research activities and trainings. But an independent organization which could take up the whole lot of subjects that are being taught in reputed institutes worldwide is lacking in India. On the other hand, if we try to segregate the various subjects and delve deep into their curricular perspectives, we get tinges of global health being taught in various institutes under different domains in India, which are undoubtedly not sufficient.

## Discussion

Health has transcended national boundaries, facilitated by increased globalization, migration of people and increased permeability of borders by wars. Control of infectious diseases has become challenging and chronic diseases are becoming more and more prevalent. It is therefore important that health professionals have sufficient preparedness to treat patients across varied disease and geographical profiles. Further, the Sustainable Development Goals, adopted in 2015, presented a major milestone in unifying efforts toward global development, thereby causing significant shift in health priorities. With increasing recognition of global health as a distinct domain, what assumes prime importance is the strengthening of existing capacity and building new capacity that can support present health related academic programs in imparting global health education. Professionals including researchers and practitioners should therefore be imparted necessary training to help them acquire a global health perspective and enable them to evaluate medical and health issues in the global context.

### Global Landscape

Globalization has led to increased pathogen flow, information, finance, business, and also migration of people within and across countries. The global health needs therefore have remained similar for populations worldwide, including countries like India. It is therefore prudent to understand how western countries have catered to the delivery of global health education. Dedicated global health courses have started to appear in the UK, Sweden, the Netherlands, USA, Finland, Germany, Canada, Australia, New Zealand, and Peru (4).

In Sweden, global health has been a part of the medical curriculum at the Karolinska Institute since 1996. It now has a Centre for Global Health (CGH), an interdisciplinary platform dedicated to improving health and achieving health equity for people worldwide, through research, education, and training. Through its CGH, the Karolinska Institute offers a 1-year Master’s Programme in Global Health that aims at providing a common knowledge base, teaching on different specific areas of global health and a research to be carried out either in Sweden or in a low- or middle-income country. The CGH also offers a PhD program in Biology of Infections and Global Health Programme and supports several interdisciplinary and online courses in global health. Postgraduate programs in global health are also offered at the Uppsala University, Umeå University and University of Gothenburg in Sweden. The Swedish Research School in Global Health is a partnership between Umeå University, Karolinska Institutet, and Lund University and provides interdisciplinary encounters through seminars, courses and workshops.

In the United Kingdom, several universities are offering BSc in global health. For example, at the Imperial College, London, the Global Health BSc is a 1-year course, launched in 2010 for undergraduates from medical and other relevant undergraduate programs. Similar full time program for undergraduates and postgraduates are also offered by several other universities such as the University of Oxford, University of Edinburgh, and University of Glasgow. Intercalated BSc Global health is offered by the University of Manchester, which is another 1-year program for medical students, designed to analyze the impact of major social, economic, political, cultural, and environmental factors on health challenges. At the University College London, the Institute for Global Health (IGH; also called UCL institute since August 2013) is an interdisciplinary collaboration working on health and development in a global context. The IGH offers yearlong global health—BSc course at undergraduate level; masters, diploma, and certificate degrees in global health at post graduate level; and also offers research degree programs such as MPhil or PhD in global health. The Global Health Education and Learning Incubator at Harvard University support innovative learning, teaching, and dialog about global challenges.

Global health also forms an important priority research area for the University of Amsterdam. The Amsterdam Institute for Global Health and Development under the University of Amsterdam is a network on research, education, training, and policy advice across disciplines. The Maastricht University and the Vrije Universiteit in the Netherlands offer 1- and 2-year Master’s Programme in Global Health, respectively.

A website review of medical schools in the United States of America (USA) conducted in 2013 indicated that 32 out of the 133 surveyed schools offered structured global health programs to students, but there was lack of uniformity and standardization across programs (5). A report by the CSIS Global Health Policy Center in 2009 analyzed the expansion of University engagement in global health in the USA and Canada to find that 41 universities had pan-university institute or centers, and 11 universities had global health programs within existing departments and divisions (6). Academic conferences, societies, consortiums, and associations have also been established internationally to coordinate the effort of global health education, research, and practice. The Consortium of Universities for Global Health (CUGH) founded in 2008 by the Bill and Melinda Gates Foundation and the Rockefeller Foundation and the Global Health Education Consortium (GHEC), another non-governmental agency founded in 1991, are few such examples which consist of faculty and health care educators dedicated to addressing global health challenges. In 2011, the merger between CUGH and GHEC was completed leading to a stronger membership base and the expansion of educational resources.

### Indian Landscape

In India, global health education has recently been garnering attention. The first and essential step for enhancing capacity in this direction is to understand the current status of the curriculum and design newer models. We therefore conducted a curricular mapping to assess the present status of global health teaching in India. Using an iterative process, we inventoried independent academic programs on global health as well as examined teaching of global health within undergraduate and postgraduate health professional education. In the following paragraphs, we analyze the current positioning of global health teaching in India and discuss the potential implications of our findings against the backdrop of growing importance of global health.

#### Fragmented Delivery of Global Health Education

An analysis of the Tables 1 and 2 shows that in India, the delivery of global health education is presently fragmented. Global health teaching is imparted either as (i) a certificate course (ii) as an online course, or (iii) as part of the topic being covered in modules of masters of public health/diploma in public health/medical tourism or in public health management courses. Moreover, it is also observed that apart from very few institutions, global health teaching is offered along with several other domains such as international health and public health but never as a single entity. There appears to be hardly any institutions which are imparting global health education in the form of a distinct educational program. Globally, in developed countries, education in global health encompasses distinct undergraduate and postgraduate level programs such as bachelors, masters, or even PhD. In contrast we see that the framework of global health education in India is yet at its nascent stage. The content of global health teaching in India caters to those such as global health issues, policies, management, globalization, affairs, and organizations. There is also no definite pattern with respect to teaching global health neither is there any focus or approach to impart it independently as a subject in our country. Areas of interest such as energy, feminism, politics, nutrition, migration, environment, and poverty at global levels do not seem to arouse much interest for studies in India. Some faculties might just include them in their discussions or lectures as a part of their liking or interest. This may be attributed to the fact that while we as a country may have undoubtedly evolved economically, but the growth has not been parallel.

#### Need for Building Greater Interest among Medical Professionals

It is often observed that to improve income standards, the student community often prefers opting for courses which warrant better returns financially after jobs. But placements of global health studies do not promise such illuminating future as far as jobs are concerned. Thus such courses in India remain limited to either being studied in parts or being taken up as online courses or in continuation with some foreign funding agencies. The topic has thus never been given enough priority that we at our levels try and initiate a separate course curriculum for it.

For health professionals to have a strong voice in health related discussions and role in addressing pressing health problems, it is important that they have good hold on the international dimensions of their subject and global health topics become integral part of the medical education (7). Exploring global health issues will also aid the general development of a health professional. Although, many research and training programs are being carried out in India, yet, they are not independent curriculums. Organizations take up such projects only in collaboration with foreign funding agencies or as part of any short-term training. Moreover, few professionals in India tend to teach global health and related issues out of their personal interest and inquisitiveness, although it does not form a part of any curriculum.

#### Lack of Focus on Global Health Education at Undergraduate and Postgraduate Level

Indian universities and schools have just begun to realize the need for introducing global health curricula. India currently has 398 medical colleges (3), but none of them have started imparting global health education to its students. The curriculum in the medical colleges of India at the undergraduate and postgraduate level lacks global perspectives of all health related issues. Details about few of the international organizations in health are covered up in Community Medicine. Even the curriculum for postgraduates in Community Medicine shares a similar picture with only some parts dedicated to global health, such as international health regulations and global networks. The column for eligibility criteria in Table 2 also indicates that there is no definite categorization or specification with respect to who can pursue the course. While mere graduation is the desired criteria in certain cases, there are also few other institutes that offer it to MBBS students only.

## Recommendations

Global health has so far been primarily defined by institutions from developed countries and in terms of their working with developing countries. Despite the fact that the burden of preventable disease is more concentrated in the middle- and low-income countries, most of the global health centers are located in high-income countries. This may be attributed to the following reasons: (i) centers in low- and middle-income countries are engaged in global health issues but under other labels. For example, several centers in low- and middle-income countries have recently been funded by the National Heart, Lung and Blood Institutes to undertake chronic disease prevention activities, though the focus seems to be on national programs of work; (ii) global health may be seen to be separated from the health needs of low- and middle-income countries which are already grappling under the pressure of many other challenging issues; (iii) strong national public health institutions help in instigating an interest in global public health among masses, which are usually lacking in LMIC (8). There is a danger that all this new energy for global health will lead to it becoming an activity developed through the lens of rich countries, ostensibly for the benefit of poor countries, but without the key ingredients of a mutually agreed collaborative endeavor (9). It is therefore important and essential that global health education emerges as a global priority for medical and health education curriculum.

The teaching curricula should cover methods and skills to describe the global health status, to compare differences in health status with a global perspective, to prioritize medical and health issues, to effectively obtain relevant and objective evidence supporting global health decision-making. Local and global data on populations can often be found in existing sources, such as various types of yearbooks published by governments and datasets from the WHO and the World Bank, most of which are available on the web. Many traditional descriptive and comparative methodologies and skills can be taught along with lessons on global epidemiology covering GIS technology for global spatial and geographic mapping. Teaching of global health needs to be integrated into broader public health curriculum which will enable students to demonstrate full array of knowledge, skills, and attitudes and be better prepared for a wide variety of global workforce positions. Further, more placement opportunities for students having taken up global health courses should be considered such that students are encouraged to take up this field professionally.

Teachings and learning can be acquired from various international bodies, non-profit organizations and agencies in the implementation of successful interventions across the world. Similar to the economic globalization, one key part to global health intervention research is to teach students how to optimize the health resource allocation with a global perspective to maximize the effect of an intervention strategy and to minimize health inequality across countries/regions and the globe thus promoting equity.

Global health education should also aim for incorporation of cross-cultural component into the training program thus necessitating the teaching of programs like economic globalization, geography, foreign languages, communication, diplomacy, sociology, cross-cultural psychology, and international health systems. A study by Bozorgmehr on global health education framework, emphasized on the territorial, trans-territorial and the supra-territorial dimensions of global health, but the framework did not specify a prescriptive catalog of topics for global health with detailed educational outcomes, since it was not a curricular proposal (10).

Development of a global health education framework should also consider building on existing strengths in the country in terms of public health delivery, leadership, systems, and training. This should also entail working through national and global collaborations to facilitate sharing of knowledge and expertise on matters related to global health teaching.

Taking into consideration the above recommendations and based on analysis of existing research, a template syllabus for global health has been proposed (Table 3). The proposed syllabus encompasses key essential elements that should become part of global health teaching and may be customized as per individual country/program needs.

**Table 3:** Proposed template syllabus for global health teaching.

Module I: Basic concepts in global health	
01.	Defining and measuring global health
02.	Global health definitions, case studies
03.	Historical origin and evolution
04.	Health systems and global health

**Module II: Understanding the key global health challenges**	

01.	Current global health status
02.	Global burden of disease
03.	Global health priorities for twenty-first century
04.	Global health at the human–animal–ecosystem interface

**Module III: Cross-cutting themes in global health and emerging trends**	

01.	Environment, climate, and migration
02.	Food, water, and sanitation
03.	Health disparities
04.	Women’s health
05.	Emerging, remerging infectious diseases
06.	NCD and injuries
07.	Maternal and child health
08.	Childhood immunization
09.	Adolescent health
10.	Neglected tropical diseases
11.	Antimicrobial resistance

**Module IV: Global health diplomacy**	

01.	Overview of global health diplomacy
02.	Global health actors and activities
03.	Global health financing
04.	Global health policy and governance
05.	Drivers of policy for global health diplomacy
06.	Globalization, trade, work, and health
07.	Foreign policy and health

**Module V: Global health security**	

01.	Global health security
02.	Pandemics and health security responses
03.	Health in complex humanitarian emergencies
04.	Humanitarian response and humanitarian dilemmas
05.	Global health equity
06.	Values in global health
07.	Toward a social justice approach to global health

**Module VI: Research, development, innovation, and technology for global health**	

01.	The environment, sustainable development, and health
02.	Universal health coverage in the context of aging
03.	Sustaining good health with equity at low cost
04.	Science and technology for global health
05.	Scaling up effective models in global health delivery

Finally, design and development of global health programs and global health teaching should encompass ethical considerations. While global health training frequently includes field experiences, these may sometimes lead to ethical challenges such as burden on the host in the resource-constrained setting; negative impact on patients, community, and trainees; unbalanced relationships among institutions and trainees; and issues related to sustainability and optimal resource utilization (11). It is therefore critical that as global health education framework is developed, the pros and cons of global health training programs are assessed. The guidelines on ethics and best practices for field-based global health training in institutions, trainees, and sponsors proposed by the Working Group on Ethics Guidelines for Global Health Training could be utilized effectively for assessment and improvement of existing programs as well as design of new programs.

## Limitations

This study only considered teaching programs in global health containing clinical or public health component and is mainly targeted for health professionals. Thus, academic programs in social and humanities stream relating to global health or international studies (social and psychological dimensions) were not included in our study purview and hence have not been described in this study. The given list may not be exhaustive owing to two reasons. First, despite our iterative search strategy, we might have omitted few programs inadvertently. Second, being a cross-sectional time-bound study, any program that got newly inducted after our search may have not been incorporated in our results. Nevertheless, we suggest undertaking similar exercise at periodic interval to have an updated scenario. In addition, the data uploaded on institute sites might not provide the exact scenario or might not be updated, thereby sometimes presenting a distorted picture, which might be a limitation for our study as a part of Internet search.

## Conclusion

We suggest that academic institutions can contribute in closing the developing country/developed country dichotomy by generating global health leaders who can address developing and developed country priorities simultaneously wherever they are based. A common forum addressing merits and demerits of all global health issues or affairs might provide an insight for what to include in the curriculum for studies on such matters. We can create more opportunities for placement of students after taking up the global health courses, so that students are instigated and inspired to take up the study as profession. A broader public health curriculum that integrates global health can be created, which will assist faculty in preparing students for a variety of global health programs. This will enable students to demonstrate full array of knowledge, skills, and attitudes and be better prepared for a wide variety of global workforce positions. Global health, as a branch of science and a discipline in the broad field of medicine, should be taught after developing a comprehensive framework with a focus on descriptive global health, global health etiology, and global health intervention in such a way that an era of global health begins where there is optimal allocation of limited resources with maximization of people’s health and the medical and other health professionals are actively taking part in shaping the global health movement.

## Author Contributions

All the authors have made a substantial contribution to the conception and design and/or the analysis and interpretation of data, drafting the article as well as revising it critically for intellectual content.

## Conflict of Interest Statement

The authors declare that the research was conducted in the absence of any commercial or financial relationships that could be construed as a potential conflict of interest.
